# Effects of Rucksack Military Accessory on Gait Dynamic Stability

**DOI:** 10.36001/ijphm.2021.v12i4.2778

**Published:** 2021-08-24

**Authors:** Seong H. Moon, Christopher W. Frames, Rahul Soangra, Thurmon E. Lockhart

**Affiliations:** 1 School of Biological and Health Systems Engineering, Arizona State University, Tempe, AZ,85281, USA; 2 Crean College of Health and Behavioral Sciences, Chapman University, Orange, CA, 92866, USA; 3 Fowler School of Engineering, Chapman University, Orange, CA, 92866, USA

## Abstract

Various factors are responsible for injuries that occur in the U.S. Army soldiers. In particular, rucksack load carriage equipment influences the stability of the lower extremities and possibly affects gait balance. The objective of this investigation was to assess the gait and local dynamic stability of the lower extremity of five subjects as they performed a simulated rucksack march on a treadmill. The Motek Gait Real-time Interactive Laboratory (GRAIL) was utilized to replicate the environment of the rucksack march. The first walking trial was without a rucksack and the second set was executed with the All-Purpose Lightweight Individual Carrying Equipment (ALICE), an older version of the rucksack, and the third set was executed with the newer rucksack version, Modular Lightweight Load Carrying Equipment (MOLLE). In this experiment, the Inertial Measurement Unit (IMU) system, Dynaport was used to measure the ambulatory data of the subject. This experiment required subjects to walk continuously for 200 seconds with a 20kg rucksack, which simulates the real rucksack march training. To determine the dynamic stability of different load carriage and normal walking condition, Local Dynamic Stability (LDS) was calculated to quantify its stability. The results presented that comparing Maximum Lyapunov Exponent (LyE) of normal walking was significantly lower compared to ALICE (P=0.000007) and MOLLE (P=0.00003), however, between ALICE and MOLLE rucksack walking showed no significant difference (P=0.441). The five subjects showed significantly improved dynamic stability when walking without a rucksack in comparison with wearing the equipment. In conclusion, we discovered wearing a rucksack result in a significant (P < 0.0001) reduction in dynamic stability.

## 1. Introduction

Load carrying equipment is used in various daily contexts, ranging from school backpacks carrying textbooks/supplies and recreational hiking backpacks to military applications.

Accordingly, the purpose of the load carriage equipment in a military context is to enhance and maximize a soldier’s performance. However, inadequate design can reduce performance and even result in injuries given the increase in mass, fatigue, and duration. Therefore, it is necessary to evaluate the effect of these supportive devices and whether they hinder the potential stability of a soldier. One of the most important pieces of equipment that is utilized in the U.S military is the rucksack, a simple form of load carriage. In the military recruitment qualification process, a rucksack march evaluates the basic physical strength and stamina of a soldier and whether they qualify for the military. Also, it is essential to indicate gait instability with various load carrying equipment to determine their effects on injuries during training (Knapik et al., 1992).

The U.S military has reported that the recommended standard rucksack weight should be less than 33kg for a soldier to perform at their optimal physical condition (Meehan, 1990). Additional equipment, such as a rifle, bulletproof vest, and Kevlar, will increase the weight demand that soldiers need to carry. Previous investigations report that during military training, 82% of the majority of accidents are the direct result of slips, trips, and falls (Okeeffe et al., 2014). Thus, the heavier the rucksack, the greater increase in gait instability. To lower the rate of musculoskeletal injuries and reinforce the rucksack, the U.S. army has replaced ALICE, an older version of rucksack built with a metal frame which was manufactured by the United States Army Support Center, with a more efficient rucksack made of a plastic frame called MOLLE, manufactured by Specialty Defense System.

To assess different load carriage conditions on gait stability, LDS was quantified with the Maximum LyE. Maximum LyE(λ) delineates the average logarithmic rate of divergence, in which a higher value means the system is unstable with a larger divergence between the nearest neighbor, and a lower value indicates that the structure of the system is more stable. The previous study indicated that fall prone subjects have higher Maximum LyE compare to healthy old and young subjects, which present that fall prone individuals have significantly lower LDS compared to healthy counterparts (Bizovska et al., 2018; Lockhart & Liu, 2008; Toebes et al., 2012).

The objective of this study was to compare normal walking condition with two different types of load carriage equipment and determine the variations of the LDS.

## 2. METHODS

### 2.1. Experimental Protocol

Five healthy male subjects were recruited from Arizona State University. The subjects’ anthropometric data were the followings: 24 ± 2.5 years of age; 178.5 ± 2.4 cm of height; 77.3 ± 19.8 kg of weight; 24.2 ± 6.0 kg/m^2^ of Body Mass Index (BMI). To reproduce the rucksack march in the laboratory environment, Motek Gait Real-time Analysis Interactive Laboratory (Motek, GRAIL, Amsterdam, Noord-Holland, the Netherlands) system was used to simulate the rucksack march. For the data assessment, a single IMU accelerometer (DynaPort MM+, Den Haag, the Netherlands) with a sampling frequency of 100 Hz was utilized to assess the gait data from the subject. The device was located at the sacrum area with elastic waistbands. All participants who participated in this study provided written consent before the beginning of data collection, which this study was approved by the Institutional Review Board (IRB: STUDY 00003645) of Arizona State University.

Prior to the data collection, subjects were asked to walk at least five minutes on the treadmill to acclimatize themselves to the treadmill, rucksack, and environment. Participant’s preferred walking velocity was determined with and without the load carriage (Yang & King, 2016). All participants walked at their preferred walking speed. Subsequently, participants carried out all walking trials for 200 seconds: First, normal walking was performed without any load carriage; second, subjects carried an established type of steel frame rucksack, ALICE with 20 kilograms of weight to simulate the real-life rucksack march; third, subjects wore the MOLLE rucksack with a carrying load of 20kg. Participants were asked to wear the U.S. army Kevlar helmet, vest, and rucksack to simulate the actual rucksack march that enlisted soldiers would perform. Appropriate rest was provided between each trial to avoid fatigue influencing the stability of walking.

### 2.2. Data analysis

The following procedure was processed to calculate the dynamic stability of each subject’s normal and load carriage data. Before proceeding into the calculation of dynamic stability, raw gait data from the IMU accelerometer was low pass filtered using zero-lag fourth order Butterworth filter to remove high frequency noise from sensor data (Yu et al., 1999). Once the data is filtered consistent gait cycles were extracted. After a consistent, steady-state speed was achieved, a threshold-based peak detection algorithm was used to identify 50 gait cycles and truncate the dataset for analysis. Then, the dataset was normalized so that each gait cycle was resampled to 100 data points each. Normalizing the gait cycle to 100 data points is a standard technique in gait analysis. To calculate the dynamic stability, the most important process is finding maximum LyE. Maximum LyE indicates the average logarithmic rate of divergence, hence, the higher value designates instability of the larger divergence between the nearest neighbors, in contrast, a lower value determines more stableness (Dingwell & Cusumano, 2000). In this computation, the Rosenstein method was utilized to calculate the maximum LyE (Rosenstein et al., 1993). To establish the proper time delay coordinate, it was determined by using average mutual information. From the plotted graph of average mutual information, the first minimum was set as the time delay. Lastly, the maximum LyE value was determined with reported time delays coordinate (10th) and embedding dimension (5th) (Packard et al., 1979; Takens, 1981). The result was utilized to determine the difference between the normal and various load carriage walking conditions which reported varied maximum LyE values.

### 2.3. Statistical analysis

To determine the most stable status from three different situations, the mean of maximum LyE was calculated for each carriage condition. To calculate the significant difference between various conditions, data were compared for all mean pairs by using post-hoc Tukey-Kramer HSD (Honestly Significant Difference) analysis. Tukey-Kramer HSD test was applied to perform an exact alpha level test of the same sample size (Kramer, 1956; Tukey & Braun, 1994). The dependent value was the maximum LyE value, and the independent value was the three load conditions, which were no load, ALICE and MOLLE. The outcome showed how the maximum LyE value differed given each condition.

## 3. Results

We found that ALICE had the highest mean LyE value of 1.31, MOLLE had the value of 1.25, and the normal walking showed a mean LyE value of 0.89, which was the lowest divergence rate among all others. This demonstrates that the load carriage and rucksack usage component have a considerable effect on dynamic stability.

**Figure 3.**
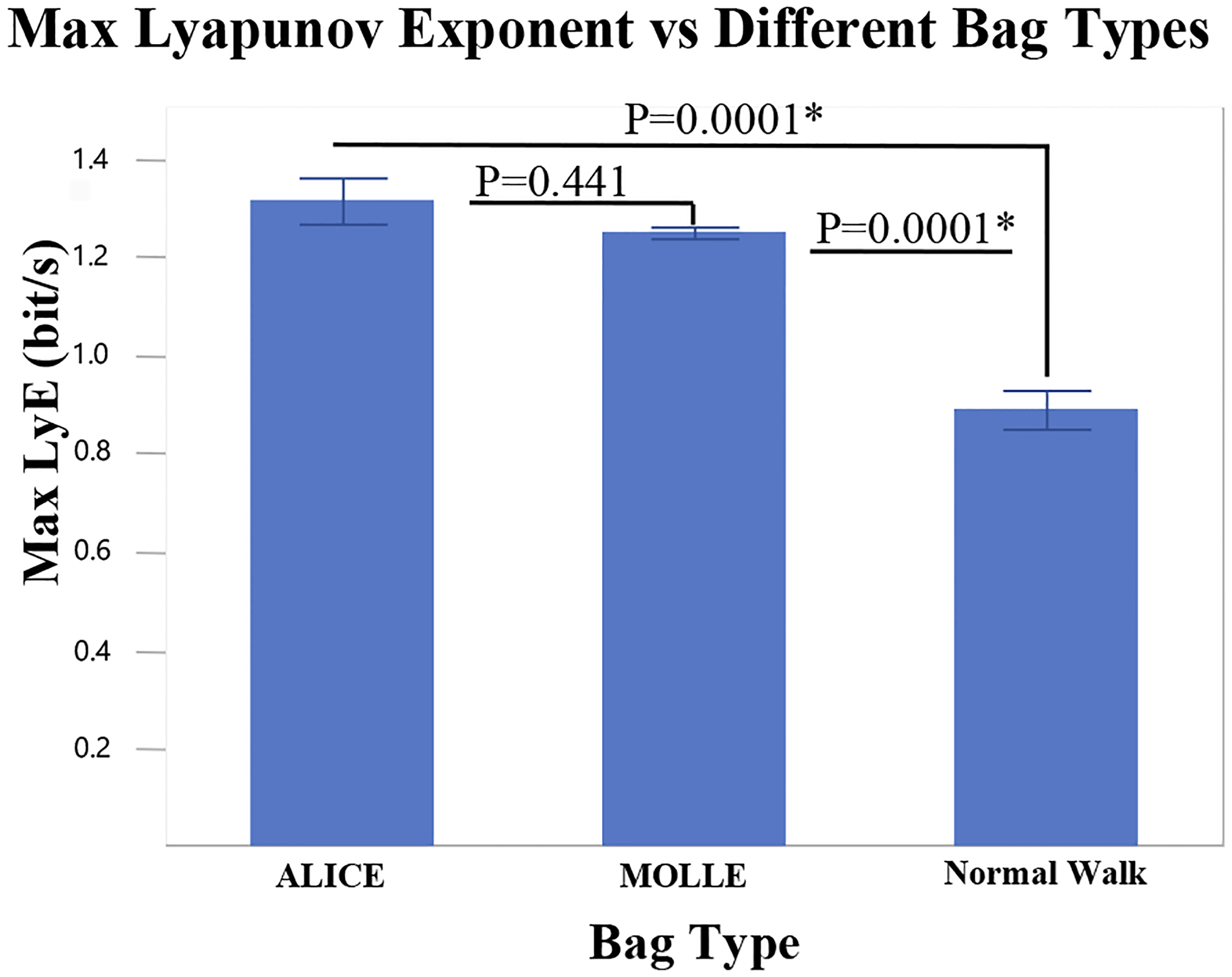
Bar graph analysis of three different walking condition effect on dynamic stability

In addition, as presented in Figure 3, the results indicated that ALICE and MOLLE do not show significant differences in maximum LyE between these walking conditions (P-value=0.441). However, when comparing the result of ALICE with Normal walk, the p-value was 0.000007; MOLLE and Normal walk’s p-value was 0.00003. Therefore, we were able to determine significant differences between the normal walking and both of the bag types, however, no significant differences were revealed amongst ALICE and MOLLE.

Moreover, as presented in Figure 3, the results indicated that ALICE and MOLLE do not show significant differences in maximum LyE between these walking conditions (P-value=0.441). However, when comparing the result of ALICE with Normal walk, the p-value was 0.000007; MOLLE and Normal walk’s p-value was 0.00003. Therefore, we were able to determine significant differences between the normal walking and both of the bag types, however, no significant differences were revealed amongst ALICE and MOLLE.

## 4. Discussion

The purpose of this study was to determine how the weight and material of the load carriage affect the dynamic stability of the subject’s gait. Previous studies reported that load carriage modifies the push-off force and generates massive fatigue affecting gait instability (Birrell & Haslam, 2010; Qu & Yeo, 2011). Considering these factors, as hypothesized, normal walking without any bag had the most stable dynamic stability, while MOLLE had the second highest dynamic stability, and ALICE was the least stable condition among the three circumstances. Compared to ALICE and MOLLE bag type walking, normal walking was found to have the lowest values of maximum LyE values. This indicates that certain physical forces applied during walking may develop significant gait instability. In addition, the maximum LyE rate comparison between material difference among the ALICE (Metal) and MOLLE (Plastic) indicated no significant difference. Therefore, whether the load carriage material is metal or plastic frames, it does not influence the dynamic stability significantly.

Despite the significant findings, there are several limitations in this study. This research was conducted with five young male people, which could be considered a low sample size. A larger and more diverse sample population would provide a more accurate and reliable translation to the larger population. Furthermore, increasing the sample size, such as 95^th^ to 5^th^ percentile male should be tested in order to acquire the normally distributed sample for dynamic stability. Moreover, different age groups and gender should be considered for the potential subject. This will increase the reliability of the data and understand how dynamic stability performance differs in another experimental group.

Understanding the limitations of the current research, future studies should consider assessing overground load carriage. This is important since practically military use of rucksacks is in varied terrain environments instead of constrained treadmill walking conditions. However, these data collected in a virtual reality environment simulated natural walking to some extent. Accordingly, investigating the overground load carriage with various circumstances, such as providing a different kind of land terrain and incline/decline sloped surfaces would provide more understanding of how dynamic stability is effect by various load carriage situations.

## 5. CONCLUSION

This study investigated the effect of load carriage and material difference on young healthy adult’s dynamic stability. The result revealed that normal walking without any load carriage had the most stable dynamic stability, and the other two bag types reported higher LyE values compared to normal walking. In addition, these two-load carriage conditions showed that there was no significant difference in dynamic stability. This implies that certain load conditions have a significant effect on dynamic stability, however, given equal weight, different material component of load carriage does not alter the subject’s dynamic stability.

## Figures and Tables

**Figure 1.**
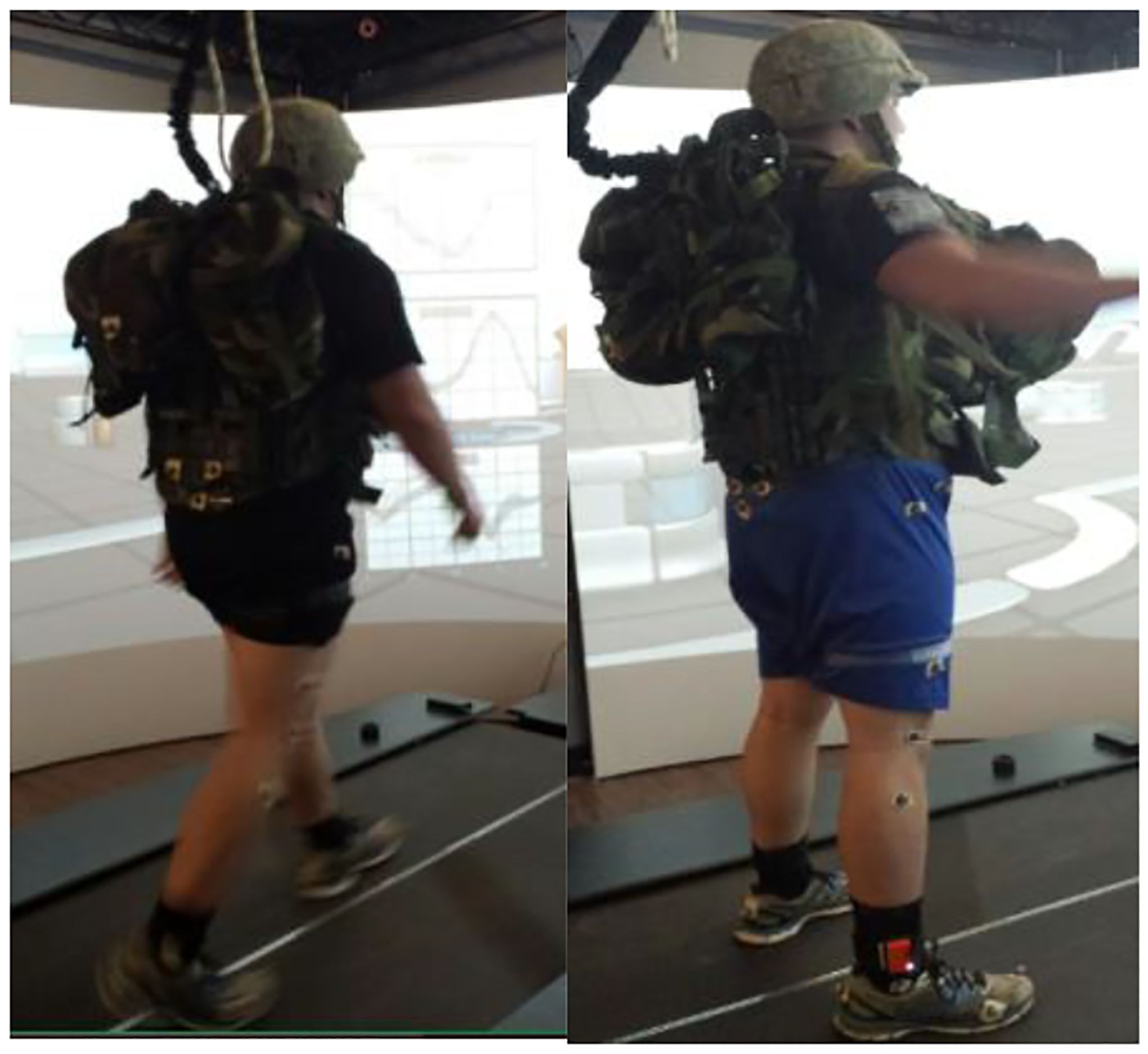
Subject Load Carriage Simulation on GRAIL system

**Figure 2.**
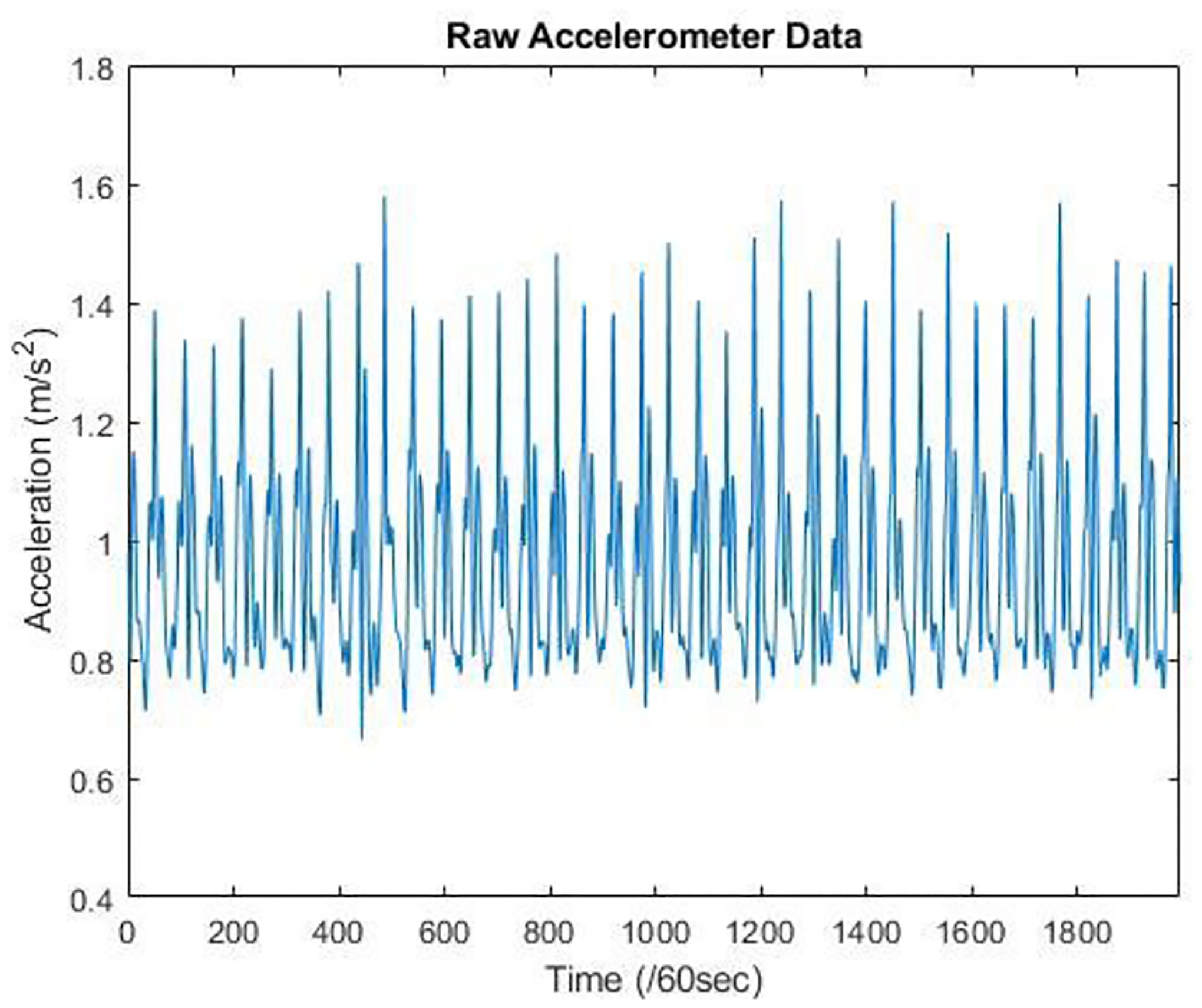
Raw Acceleration Data measured from Inertial Measurement Unit

**Table 1.** Each subject’s anthropometric data

	Subject Anthropometry				
ID	Age (years)	Height (cm)	Weight (kg)	BMI (kg/m^2^)	Gender
S001	24	180.8	79.2	24.23	Male
S002	22	177	48.65	15.53	Male
S003	22	179.5	104.25	32.36	Male
S004	28	175	75	24.49	Male
S005	24	180	79.4	24.51	Male

**Table 2.** Maximum Lyapunov Exponent for Each Subject with Different Load Carriage Condition

	Maximum LyE (λ) bits/s		
ID	Normal Walk	ALICE	MOLLE
S001	0.82	1.40	1.29
S002	1.01	1.31	1.24
S003	0.79	1.14	1.22
S004	0.93	1.34	1.24
S005	0.91	1.39	1.26

## Nomenclature

ALICE: All-Purpose Lightweight Individual Carrying Equipment
MOLLE: Modular Lightweight Load Carrying Equipment
LDS: Local Dynamic Stability
LyE: Lyapunov Exponent

## References

[R1] Ahn J (2011). Feasibility of Novel Gait Training with Robotic Assistance: Dynamic Entrainment to Mechanical Perturbation to the Ankle. ProQuest Dissertations and Theses. http://ezproxy.ithaca.edu:2048/login?url=https://search.proquest.com/docview/916764118?accountid=11644%0Ahttp://ithaca-primo.hosted.exlibrisgroup.com/openurl/01ITHACACOL/01ITHACACOL_SP?rft.genre=dissertations+%26+theses&rft.atitle=&rft.jtitle=&rft.btitle=

[R2] Birrell SA, & Haslam RA (2010). The effect of load distribution within military load carriage systems on the kinetics of human gait. Applied Ergonomics, 41(4), 585–590. 10.1016/j.apergo.2009.12.004 20060096

[R3] Bizovska L, Svoboda Z, Janura M, Bisi MC, & Vuillerme N (2018). Local dynamic stability during gait for predicting falls in elderly people: A one-year prospective study. PLoS ONE, 13(5), 1–11. 10.1371/journal.pone.0197091 PMC5944953 29746520

[R4] Dingwell JB, & Cusumano JP (2000). Nonlinear time series analysis of normal and pathological human walking. Chaos, 10(4), 848–863. 10.1063/1.1324008 12779434

[R5] Knapik J, Reynolds K, Staab J, Vogel JA, & Jones B (1992). Injuries associated with strenuous road marching. Military Medicine, 157(2), 64–67. 10.1093/milmed/157.2.64 1603388

[R6] Kramer CY (1956). Extension of Multiple Range Tests to Group Means with Unequal Numbers of Replications. Biometrics. 10.2307/3001469

[R7] Lockhart TE, & Liu J (2008). Differentiating fall-prone and healthy adults using local dynamic stability. Ergonomics, 51(12), 1860–1872. 10.1080/00140130802567079 19034782 PMC2892176

[R8] Meehan WI (1990). ARMY Field Manual.

[R9] Okeeffe T, Hrymak V, & O’Sullivan L (2014). AN ANALYSIS OF MUSCULOSKELETAL ACCIDENTS IN OPERATIONAL MILITARY TRAINING IN THE DEFENCE FORCES. 1.

[R10] Packard NH, Crutchfield JP, Farmer JD, & Shaw RS (1979). Geomerty from a Time Series. Physcial Review Letters, 45(9), 725–728.

[R11] Qu X, & Yeo JC (2011). Effects of load carriage and fatigue on gait characteristics. Journal of Biomechanics, 44(7), 1259–1263. 10.1016/j.jbiomech.2011.02.016 21397234

[R12] Rosenstein MT, Collins JJ, & De Luca CJ (1993). A practical method for calculating largest Lyapunov exponents from small data sets. Physica D, 65(1–2), 117–134. 10.1016/0167-2789(93)90009-P

[R13] Takens F (1981). Detecting strange attractors in turbulence. Springer, 10.1007/BFb0091924. http://www.springer.com/gp/book/9783540111719

[R14] Toebes MJP, Hoozemans MJM, Furrer R, Dekker J, & Van Dieën JH (2012). Local dynamic stability and variability of gait are associated with fall history in elderly subjects. Gait and Posture, 36(3), 527–531. 10.1016/j.gaitpost.2012.05.016 22748312

[R15] Tukey JW, & Braun HI (1994). The Collected Works of John W. Tukey, Volume VIII: Multiple Comparisons. Journal of the American Statistical Association. 10.2307/2291036

[R16] Weiss A, Herman T, Giladi N, & Hausdorff JM (2014). Objective assessment of fall risk in Parkinson’s disease using a body-fixed sensor worn for 3 days. PLoS ONE, 9(5). 10.1371/journal.pone.0096675 PMC4011791 24801889

[R17] Yang F, & King GA (2016). Dynamic gait stability of treadmill versus overground walking in young adults. Journal of Electromyography and Kinesiology, 31, 81–87. 10.1016/j.jelekin.2016.09.004 27694060

[R18] Yu B, Gabriel D, Noble L, & An KN (1999). Estimate of the optimum cutoff frequency for the Butterworth low-pass digital filter. Journal of Applied Biomechanics, 15(3), 318–329. 10.1123/jab.15.3.318

